# Evaluating hand hygiene knowledge, attitudes, and practices among healthcare workers in post-pandemic H1N1 influenza control: a cross-sectional study from China

**DOI:** 10.3389/fpubh.2024.1432445

**Published:** 2024-09-27

**Authors:** Qin Hong, Yuling Xu

**Affiliations:** Department of Respiratory and Critical Care Medicine, The Central Hospital of Enshi Prefecture Tujia and Miao Autonomous Prefecture, Enshi, China

**Keywords:** hand hygiene, healthcare-associated infections (HAIs), healthcare workers, infection prevention, influenza

## Abstract

**Background:**

This study evaluates the knowledge, attitudes, and practices (KAP) of hand hygiene among healthcare workers, crucial for preventing healthcare-associated infections (HAIs) in medical facilities.

**Methodology:**

This cross-sectional study assessed hand hygiene KAP among healthcare workers across various settings in Hubei, China utilizing a stratified random sampling approach from, December 25, 2023-to-April 25, 2024. A bilingual electronic survey, adapted from validated tools, was disseminated via email and social media to ensure a broad reach. Participants included diverse healthcare professionals who met specific inclusion criteria. Responses were analyzed using R software, employing descriptive and inferential statistics to identify key predictors of hand hygiene behavior and to confirm the reliability of the survey instrument.

**Results:**

The survey of 2,265 healthcare workers revealed that 77% demonstrated comprehensive knowledge of hand hygiene, 80% exhibited positive attitudes, and 94% practiced effective hand hygiene. Notable findings include a significant understanding of hand hygiene’s role in preventing respiratory illnesses (58%) and HAIs (41% agreed, 39% unsure). High compliance in practices like washing hands for at least 20 s was evident (84%), though gaps in confidence about hand hygiene techniques were noted (33% confident, 56% unsure). Binary logistic regression analysis indicated that younger healthcare workers (21–30 years) were more likely to exhibit both knowledge (OR = 7.4, 95% CI = 1.44–136, *p* = 0.059) and positive attitudes (OR = 4.48, 95% CI = 1.73–11.8, *p* < 0.001) compared to other age groups. Significant associations were found between higher income levels and positive attitudes toward hand hygiene (OR for ≥80,000 = 3.19, 95% CI = 2.05–5.02, *p* < 0.001), and between knowledge and practices, suggesting that well-informed individuals are more likely to adhere to recommended practices.

**Conclusion:**

The findings reveal robust hand hygiene knowledge but uncover critical confidence gaps among healthcare workers, urging immediate, targeted educational interventions to fortify adherence and prevent infection outbreaks.

## 1 Introduction

Influenza is a major global health concern due to its rapid mutation and the ability to cause widespread seasonal epidemics (1, 2). Annually, influenza affects 5–15% of the global population, resulting in 290,000 to 650,000 deaths, which surpasses fatalities from all other vaccine-preventable diseases combined (3, 4). The virus can be transmitted by infected individuals up to 24 h before symptom onset. Hospitalized patients, often with compromised health, are at increased risk of infection, which can lead to significant hospital outbreaks and even facility closures due to its short incubation period, mutation propensity, and efficient aerosol transmission (5, 6). In Honduras, the impact of influenza is particularly pronounced, with notable rates of mortality, hospitalization, and incidence in 2017. Influenza, along with pneumonia, represents a significant cause of mortality, accounting for 3.7% of all deaths in the country (4, 7, 8).

Seasonal influenza vaccination remains the most effective method to prevent the virus and its severe outcomes. Global health authorities, including the World Health Organization (WHO) and the U.S. Advisory Committee on Immunization Practices, recommend annual vaccination for healthcare workers (5, 8). This prioritization aims to shield them from the virus, prevent its spread to vulnerable populations, and maintain healthcare operations during outbreaks (9, 10). Vaccine effectiveness ranges from 40 to 60% in years when the vaccine aligns well with circulating strains, with antibody protection lasting up to 6–8 months (6, 11, 12).

Hand hygiene is a pivotal intervention in the control of influenza virus transmission. The WHO and the Centers for Disease Control and Prevention (CDC) underscore the importance of effective hand hygiene practices to mitigate the spread of infectious agents (12–14). The relevance of these practices intensifies during the influenza season when the incidence of viral infections escalates considerably. Understanding the community’s knowledge, attitudes, and practices (KAP) regarding hand hygiene during the influenza season is crucial for developing targeted public health interventions. This introduction delineates the foundational aspects of the KAP framework, contextualizes it within the scope of influenza prevention, and elucidates the interdependencies of these elements in fostering effective hand hygiene behaviors.

Knowledge about the modes of influenza transmission and the effectiveness of hand hygiene in preventing infection is fundamental to compliance with recommended practices. Previous studies have demonstrated that an elevated level of awareness correlates positively with adherence to hand hygiene guidelines (15). However, gaps in public knowledge often persist, particularly in distinguishing the specifics of hand hygiene, such as the appropriate technique and duration of handwashing or the use of hand sanitizers (16, 17).

Attitudes toward hand hygiene practices during the influenza season significantly influence behavior patterns. A positive attitude toward hand hygiene, including the belief in its effectiveness and the perceived severity of contracting influenza, often predicts a higher commitment to regular handwashing (18, 19). Conversely, misconceptions or underestimation of the influenza virus’s impact can lead to complacency and inadequate hand hygiene practices. The actual practices of hand hygiene encompass a range of behaviors from handwashing with soap and water to the use of alcohol-based hand sanitizers (20, 21). The consistency, technique, and context of these practices vary widely among different populations. Observational studies have revealed that even among healthcare professionals, adherence to optimal hand hygiene protocols is not universally consistent (22–24).

The interaction between KAP is complex and dynamic. Enhanced knowledge can lead to more favorable attitudes, which in turn can encourage better practices. However, this linear progression is not always observed, as social, cultural, and individual factors May also play significant roles (24). For instance, even with high knowledge and positive attitudes, practical barriers such as lack of access to handwashing facilities can impede the adoption of recommended practices (8, 14). Therefore, current research examined the KAP of healthcare professionals concerning hand hygiene to prevent influenza, highlighting the importance of consistent and proper hand hygiene measures during the influenza season. Insight into these factors is critical for developing public health strategies that improve adherence to hand hygiene protocols, thereby decreasing both the spread and severity of influenza.

## 2 Methodology

### 2.1 Study design

The current investigation adopted a cross-sectional study designed based on previous studies (10, 14, 25–27) to evaluate the understanding, attitudes, and practices concerning hand hygiene among healthcare workers in various medical settings across Hubei, China from December 25, 2023-to-April 25, 2024. A comprehensive electronic survey, developed based on established instruments, was disseminated through email links and social media platforms to contacts in various hospital staff, who then facilitated its distribution among potential participants. The study utilized a stratified random sampling method to ensure diverse representation among healthcare professionals, including doctors, nurses, and support staff across multiple regions. The survey was made available in both Mandarin and English to cater to the linguistic preferences of the respondents. It included detailed questions on demographic characteristics, knowledge of hand hygiene protocols, attitudes toward hand sanitation, and reported hand hygiene behaviors.

### 2.2 Inclusion and exclusion criteria

Participants included healthcare workers—doctors, nurses, and auxiliary staff—actively employed in ICU settings who provided informed consent and were proficient in either Mandarin or English. The inclusion criteria were broad to capture a diverse range of insights, while exclusion criteria limited participation to non-healthcare personnel, individuals under the age of 18, and those who submitted incomplete responses.

### 2.3 Measurement of attributes

The knowledge domain consisted of 16 questions that evaluated the respondents’ understanding of key aspects of hand hygiene, such as the effectiveness of alcohol-based hand sanitizers, the relationship between improper hand hygiene and healthcare-associated infections (HAIs), and the impact of hand hygiene on the spread of respiratory illnesses. Each question was designed to measure the awareness and comprehension levels of the respondents, with options “Yes,” “No,” and “Not Sure.” Correct responses were awarded one point each, allowing for a maximum score of 16. A threshold of 50% (8 points) was set to differentiate between adequate and inadequate knowledge levels.

The attitude domain comprised 12 items aimed at gaging the personal beliefs and perceived importance of hand hygiene among the participants. This section probed respondents’ confidence in their hand hygiene knowledge, their views on the necessity of strict compliance with hand hygiene protocols, and their opinion on the adequacy of current hand hygiene policies. Responses were categorized as “Yes,” “No,” or “Not Sure,” with a scoring system where “Yes” indicated a positive attitude (one point per positive response). A cumulative score of 6 or more was used to distinguish between positive and negative attitudes toward hand hygiene.

The practices section of the survey included six questions focused on actual hand hygiene behaviors, such as adherence to handwashing protocols between patient contacts, participation in hand hygiene training, and usage of hand sanitizers when soap and water are not available. This segment aimed to assess the practical implementation of hand hygiene practices, with “Yes,” “No,” and “Not Sure” responses. Each “Yes” response, indicating compliance with best practices, earned one point. A cutoff of three points was employed to classify respondents into “good” or “poor” practice categories.

### 2.4 Statistical analysis and quality control

To ensure the reliability and validity of the survey instrument, we undertook a comprehensive, multi-step validation process. This process began with the adaptation of items from previously validated tools, carefully tailored to address the specific context of post-pandemic hand hygiene practices in healthcare settings. Content validity was rigorously assessed by a panel of infection control experts, who meticulously evaluated the relevance and clarity of each item. Following this, a pilot test involving 50 healthcare workers was conducted to identify and rectify any ambiguities or comprehension issues. The internal consistency of the survey was confirmed by calculating Cronbach’s alpha, which yielded a coefficient of 0.85, indicating a high level of reliability. To further ensure the survey’s robustness, construct validity was examined through exploratory factor analysis, which validated the survey’s ability to accurately measure the intended constructs.

Once the survey data was collected, it was compiled in an Excel database and subsequently analyzed using R software (version 4.3.3), selected for its advanced data management and analytical capabilities. The preliminary data analysis involved meticulous cleaning and validation using functions from the dplyr and tidyr packages, ensuring the integrity of the dataset. Descriptive statistics provided an essential foundation for understanding the data distribution. To identify significant associations and predictors of hand hygiene practices, we employed bivariate and multivariate analyses. Specifically, Fisher’s exact test and Pearson’s Chi-squared test were used to assess relationships between categorical variables, while logistic regression analyses were conducted to determine the influence of various factors on hand hygiene behaviors. The results were presented in terms of odds ratios and confidence intervals, emphasizing both the statistical significance and the practical implications of our findings.

## 3 Results

The survey involved 2,265 healthcare workers, focusing on their hand hygiene KAP, alongside detailed demographic data. Predominantly younger, 55% of participants were aged 21–30, with 30% between 51 and 60 years, showing a skewed age distribution. The demographic profile was chiefly female (66%) and single (57%). A notable 69% of respondents came from family units of four or more. Employment was predominantly hospital-based (69%), with considerable segments in private practice and clinics. About 58% preferred not to disclose their income, indicating privacy concerns. Employment data showed 50% as permanent employees and 45% on contracts, suggesting diverse job security. The distribution of roles within healthcare revealed 51% technicians and 21% nurses, important for assessing hand hygiene compliance. Notably, 77% demonstrated sound hand hygiene knowledge, 80% held positive attitudes, and 94% practiced good hand hygiene. These statistics underscore the robust awareness and execution of hand hygiene, critical for infection control, and inform targeted interventions to enhance hand hygiene practices among healthcare workers, as shown in Table 1.

**Table 1:** Demographic variables of study participants.

Variable	*N* = 2,265
What is your age?	
≥ 61	19 (0.8%)
21–30	1,244 (55%)
31–40	316 (14%)
41–50	10 (0.4%)
51–60	676 (30%)
What is your gender?	
Female	1,489 (66%)
Male	776 (34%)
What is your marital status?	
Divorced	34 (1.5%)
Married	942 (42%)
Single	1,289 (57%)
How many members do you have in your family?	
≤ 2	115 (5.1%)
≥ 4	1,560 (69%)
3	590 (26%)
What type of institution do you currently work in?	
Clinic	128 (5.7%)
Hospital	1,557 (69%)
Private Practice	580 (26%)
What is your monthly household income?	
≤ 50,000	299 (13%)
≥ 80,000	368 (16%)
51,000-80,000	273 (12%)
Prefer Not to disclose	1,325 (58%)
What is your Employment Status?	
Contract	1,010 (45%)
Permanent Employee	1,127 (50%)
Visiting	128 (5.7%)
What is your role in healthcare setting?	
Administration	174 (7.7%)
Nurse	466 (21%)
Other	258 (11%)
Physician	220 (9.7%)
Technician	1,147 (51%)
Knowledge	
Knowledgeable	1,735 (77%)
Not Knowledgeable	530 (23%)
Attitude	
Negative Attitude	447 (20%)
Positive Attitude	1,818 (80%)
Practice	
Good Practice	2,122 (94%)
Poor Practice	143 (6.3%)
n (%)	

### 3.1 Knowledge assessment

**Figure 1:**
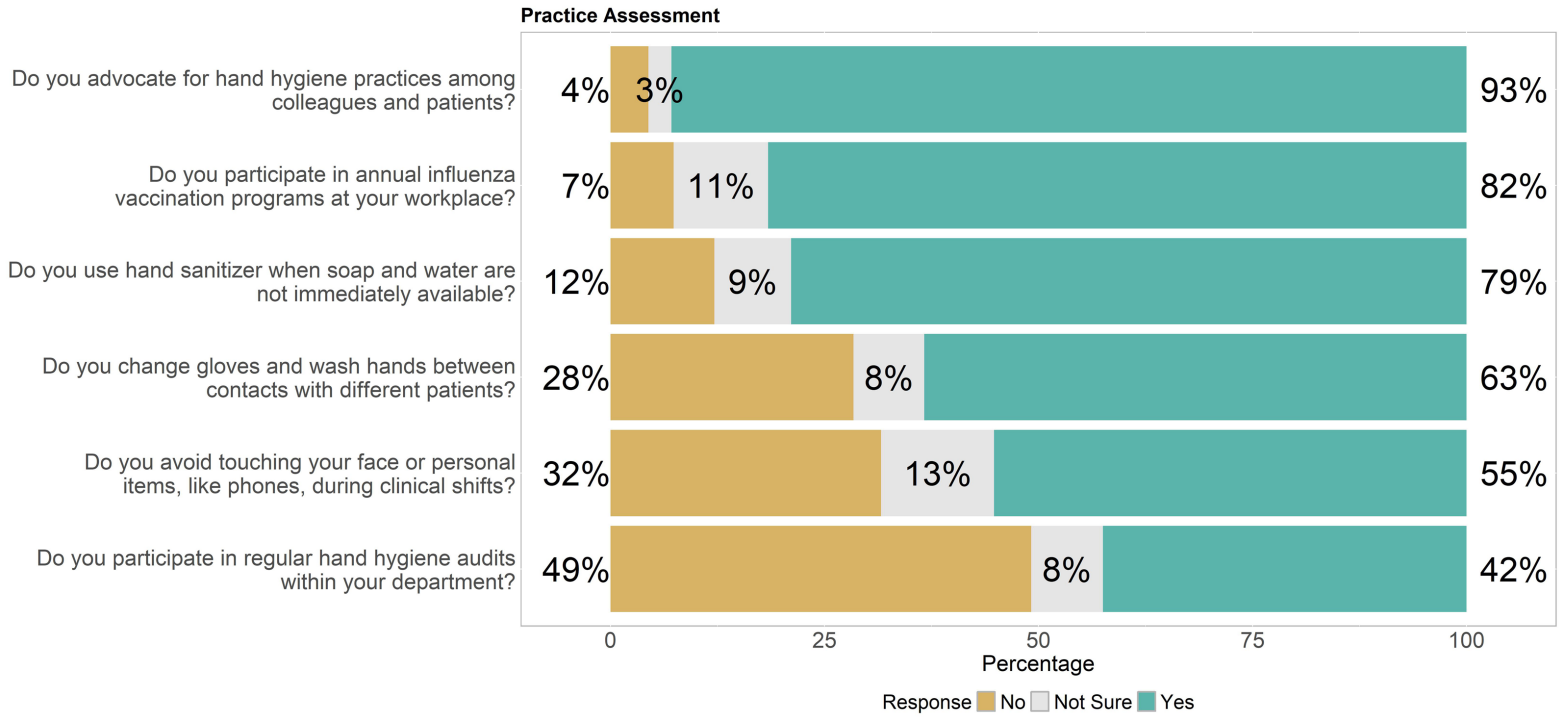
Distribution of hand hygiene knowledge levels among healthcare workers, highlighting knowledge gaps.

Responses from healthcare workers regarding their knowledge and practices of hand hygiene, structured into “No,” “Not Sure,” and “Yes” options, showed that 62% recognized alcohol-based hand sanitizers as a viable alternative when soap and water were unavailable. However, opinions were split on whether improper hygiene leads to HAIs —41% agreed, while 39% were unsure, highlighting a need for further education. Similarly, 58% acknowledged hand hygiene’s role in curbing the spread of non-influenza respiratory illnesses, though 36% remained uncertain. A significant 66% understood the importance of hand hygiene even with clean-looking hands. High compliance (73%) was seen in washing hands after touching pets or seemingly clean patient-care equipment, and 59% adhered to using sanitizers with at least 60% alcohol. Notably, the practice of washing hands for a minimum of 20 s to effectively remove germs was strongly supported by 84% of participants, underscoring a crucial, widely accepted preventive measure. This data collectively pointed to a solid foundation of good hygiene practices among healthcare workers, albeit interspersed with areas where clarity and reinforcement were needed, as shown in Figure 1.

### 3.2 Attitude assessment

**Figure 2:**
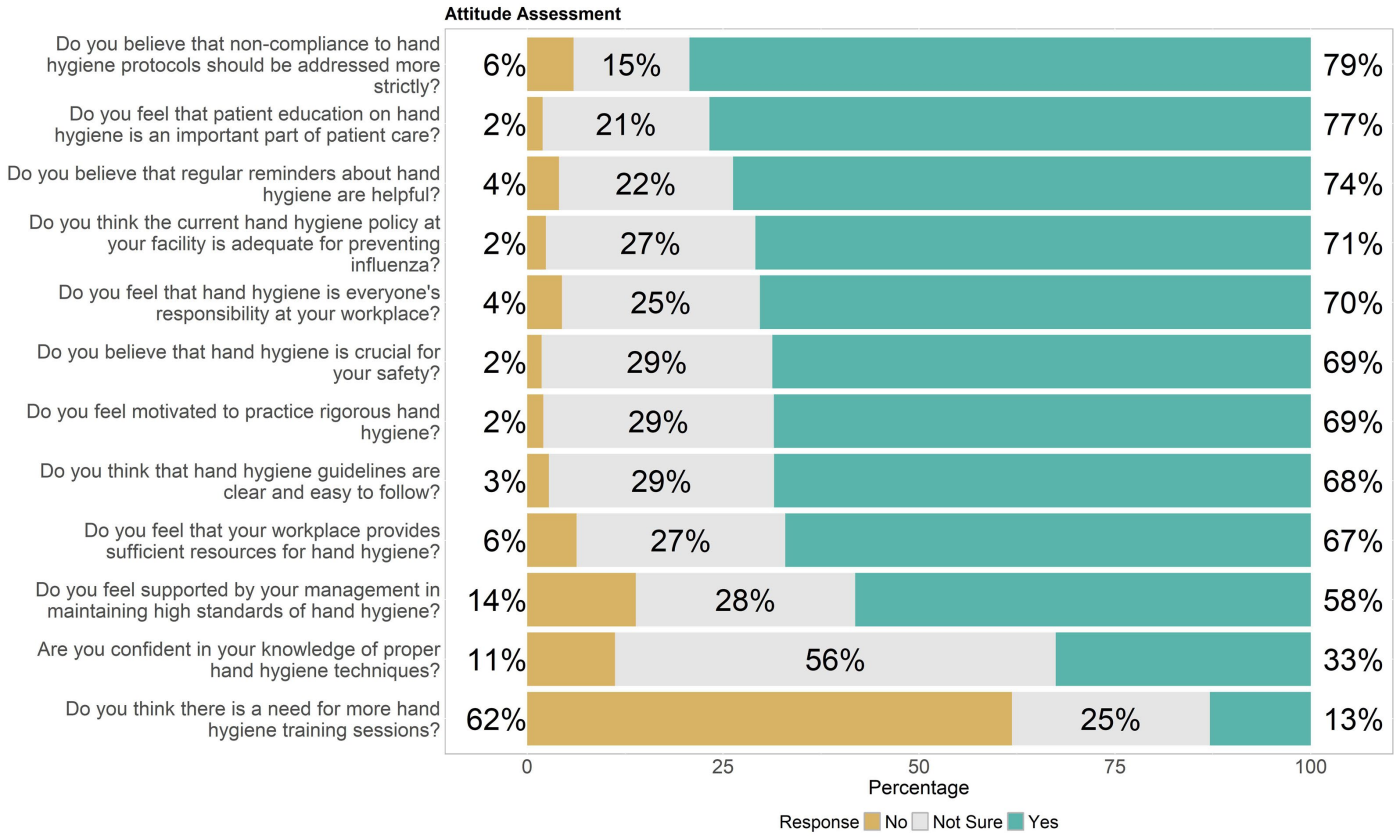
Distribution of attitude of healthcare workers toward hand hygiene, highlighting knowledge gaps.

Responses from healthcare workers about their attitudes toward hand hygiene were segmented into “No,” “Not Sure,” and “Yes” answers, showed a mixed response regarding confidence in knowing proper hand hygiene techniques—33% were confident, yet 56% remained unsure, suggesting a gap in knowledge or confidence. The majority viewed hand hygiene as vital for safety (69%) and believed in stricter enforcement of protocols (79%), indicating a strong consensus on its importance for preventing infections. About 74% found regular reminders helpful, supporting ongoing educational efforts. While 69% felt motivated to maintain rigorous hand hygiene, only 58% felt supported by management in adhering to these standards, pointing to potential areas for improvement in workplace support systems. A robust 70% agreed that hand hygiene responsibility was shared among all workplace members, and 77% recognized the role of patient education in hand hygiene as crucial. Despite strong adherence to guidelines, only 67% believed their workplace provided sufficient resources for proper hand hygiene, and 68% agreed that the guidelines were clear and easy to follow. There was a significant belief (71%) in the adequacy of current policies to prevent influenza, yet a substantial 62% saw a need for more training sessions, indicating a desire for enhanced education and resources to better implement hand hygiene practices effectively, as shown in Figure 2.

### 3.3 Practice assessment

**Figure 3:**
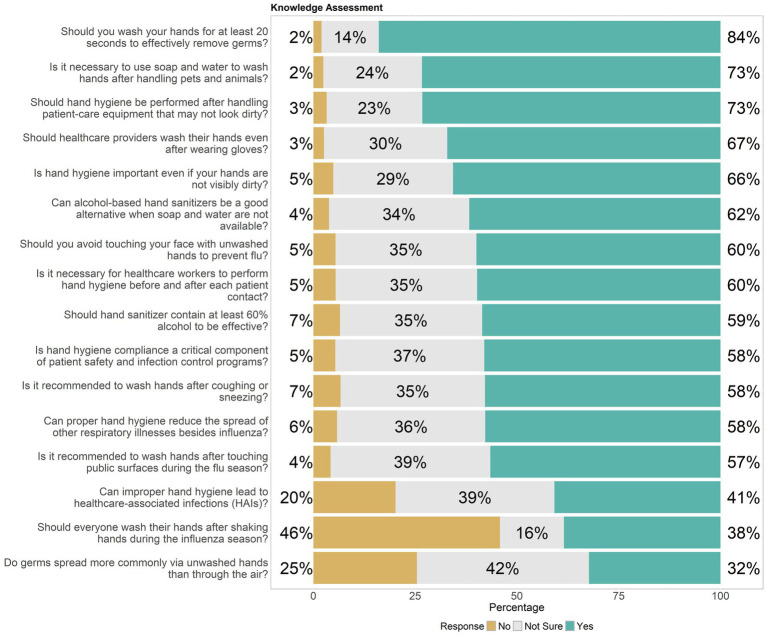
Distribution of hand hygiene practice among healthcare workers, highlighting knowledge gaps.

Healthcare workers’ responses regarding their practical engagement in hand hygiene measures, were delineated into “No,” “Not Sure,” and “Yes” categories. An overwhelming 93% advocated for hand hygiene among colleagues and patients, highlighting a strong commitment to promoting hygiene practices. About 55% managed to avoid touching their face or personal items during clinical shifts, though 32% still engaged in these potentially contaminating behaviors. In terms of procedural hygiene, 63% consistently changed gloves and washed hands between different patient contacts, but 28% did not, indicating room for improvement. A high participation rate in annual influenza vaccination programs stood at 82%, reflecting a proactive stance toward infection prevention. However, participation in regular hand hygiene audits was less frequent, with only 42% engaging regularly and 49% not participating, suggesting a need for more consistent and mandatory audits within healthcare facilities. The use of hand sanitizer as an alternative when soap and water were not available was affirmed by 79%, underlining its acceptance as a vital hygiene tool, as shown in Figure 3.

### 3.4 Binary logistic regression analysis

#### 3.4.1 Knowledge

The statistical analysis of hand hygiene knowledge across various demographic and professional variables among 2,265. The findings indicated that knowledge varied across age groups, with younger adults aged 21–30 demonstrating an odds ratio (OR) of 7.4 (95% CI = 1.44, 136, *p*-value = 0.059), suggesting a stronger likelihood of being knowledgeable. In contrast, older adults aged 51–60 showed less influence on knowledge levels with an OR of 8.42 (95% CI = 1.61, 155, *p*-value = 0.058).

Gender appeared to moderately influence knowledge, with males showing a slightly lower likelihood of being knowledgeable compared to females (OR = 1.04, 95% CI = 0.84, 1.29, *p*-value = 0.7). Marital status revealed significant discrepancies; single individuals were less likely to be knowledgeable than married ones, with an OR of 2.16 (95% CI = 0.87, 6.22, *p*-value <0.001). Family size impacted knowledge, especially in larger households, where the knowledge level was lower (OR for ≥4 members = 0.68, 95% CI = 0.42, 1.12, *p*-value = 0.009). Income levels showed a strong association; individuals earning more than 80,000 were less likely to be knowledgeable (OR = 0.64, 95% CI = 0.44, 0.92, *p*-value <0.001). Employment status correlated with knowledge; permanent employees were more likely to be knowledgeable than contract workers (OR = 0.59, 95% CI = 0.46, 0.76, *p*-value <0.001). The role within the healthcare setting did not significantly affect knowledge, as seen in the similar odds ratios across various roles. However, attitude toward hand hygiene showed a strong link with knowledge levels; those with a positive attitude were significantly more likely to be knowledgeable (OR = 0.24, 95% CI = 0.19, 0.30, *p*-value <0.001). Practice correlated positively with knowledge, with good practices associated with higher knowledge levels (OR = 1.24, 95% CI = 0.81, 1.85, *p*-value = 0.3), as shown in Table 2.

**Table 2:** Binary logistic regression analysis for knowledge assessment.

					Binary logistic regression			
Variable	N^1^	Knowledgeable, *N* = 1,735	Not-knowledgeable, *N* = 530	*p*-value^2^	Coefficient	Odds ratio	95% CI	*p*-value
What is your age?	2,265			0.059				0.058
≥ 61		18 (1.0%)	1 (0.2%)		—	—	—	
21–30		929 (54%)	315 (59%)		2	7.4	1.44, 136	
31–40		249 (14%)	67 (13%)		2.2	9.26	1.77, 171	
41–50		9 (0.5%)	1 (0.2%)		1.6	4.94	0.17, 143	
51–60		530 (31%)	146 (28%)		2.1	8.42	1.61, 155	
What is your gender?	2,265			0.3				0.7
Female		1,151 (66%)	338 (64%)		—	—	—	
Male		584 (34%)	192 (36%)		0.04	1.04	0.84, 1.29	
What is your marital status?	2,265			<0.001				<0.001
Divorced		28 (1.6%)	6 (1.1%)		—	—	—	
Married		759 (44%)	183 (35%)		0.29	1.34	0.55, 3.82	
Single		948 (55%)	341 (64%)		0.77	2.16	0.87, 6.22	
How many members do you have in your family?	2,265			<0.001				0.009
≤ 2		77 (4.4%)	38 (7.2%)		—	—	—	
≥ 4		1,176 (68%)	384 (72%)		−0.38	0.68	0.42, 1.12	
3		482 (28%)	108 (20%)		−0.72	0.49	0.29, 0.82	
What type of institution do you currently work in?	2,265			0.5				0.7
Clinic		100 (5.8%)	28 (5.3%)		—	—	—	
Hospital		1,181 (68%)	376 (71%)		0.23	1.25	0.76, 2.15	
Private Practice		454 (26%)	126 (24%)		0.23	1.25	0.73, 2.21	
What is your monthly household income?	2,265			<0.001				<0.001
≤ 50,000		190 (11%)	109 (21%)		—	—	—	
≥ 80,000		290 (17%)	78 (15%)		−0.45	0.64	0.44, 0.92	
51,000-80,000		216 (12%)	57 (11%)		−0.63	0.53	0.36, 0.79	
Prefer Not to disclose		1,039 (60%)	286 (54%)		−0.63	0.53	0.40, 0.71	
What is your Employment Status?	2,265			<0.001				<0.001
Contract		728 (42%)	282 (53%)		—	—	—	
Permanent Employee		913 (53%)	214 (40%)		−0.52	0.59	0.46, 0.76	
Visiting		94 (5.4%)	34 (6.4%)		0	1	0.63, 1.57	
What is your role in the Healthcare setting?	2,265			>0.9				0.2
Administration		131 (7.6%)	43 (8.1%)		—	—	—	
Nurse		353 (20%)	113 (21%)		0.34	1.4	0.90, 2.22	
Other		202 (12%)	56 (11%)		0.15	1.16	0.71, 1.91	
Physician		166 (9.6%)	54 (10%)		0.1	1.11	0.67, 1.83	
Technician		883 (51%)	264 (50%)		−0.01	0.99	0.67, 1.49	
Attitude	2,265			<0.001				<0.001
Negative Attitude		237 (14%)	210 (40%)		—	—	—	
Positive Attitude		1,498 (86%)	320 (60%)		−1.4	0.24	0.19, 0.30	
Practice	2,265			0.4				0.3
Good Practice		1,630 (94%)	492 (93%)		—	—	—	
Poor Practice		105 (6.1%)	38 (7.2%)		0.21	1.24	0.81, 1.85	

1 n (%).

2 Fisher’s exact test; Pearson’s Chi-squared test.

#### 3.4.2 Attitude

Attitude showed distinct variations across different demographic and professional backgrounds. Age had a considerable impact on attitudes toward hand hygiene, with significant differences observed. Younger healthcare workers aged 21–30 demonstrated a strong inclination toward positive attitudes, as evidenced by an odds ratio (OR) of 4.48 (95% CI = 1.73, 11.8, *p*-value <0.001). Similarly, middle-aged workers (31–40 years) tended positive attitudes with an OR of 7.19 (95% CI = 2.66, 19.7, *p*-value <0.001).

Gender differences were relatively marginal but notable; males were slightly less likely to hold positive attitudes compared to females, with an OR of 0.84 (95% CI = 0.66, 1.05, *p*-value = 0.13). Marital status did not show a substantial divergence in attitude, with single individuals marginally more likely to have positive attitudes compared to married ones (OR = 2.57, 95% CI = 1.06, 5.80, *p*-value = 0.1). Household size correlated with attitude, where individuals from larger households (≥4 members) were more likely to have a positive attitude (OR = 1.92, 95% CI = 1.16, 3.14, *p*-value = 0.012). The types of institutions did not significantly impact attitudes, indicating uniformity of perspectives across different work settings.

Income levels were a strong predictor of attitudes toward hand hygiene; those earning less than 50,000 exhibited fewer positive attitudes compared to higher earners (OR for ≥80,000 = 3.19, 95% CI = 2.05, 5.02, p-value <0.001). Employment status showed no significant effects on attitudes, suggesting that job security or contract terms did not markedly influence these views. In the healthcare setting, roles did not significantly influence attitudes, indicating that whether one is a nurse, physician, or technician did not drastically change their perspective on hand hygiene. However, knowledge strongly correlated with attitudes; more knowledgeable individuals were significantly more likely to hold positive attitudes (OR = 0.24, 95% CI = 0.19, 0.30, *p*-value <0.001), as shown in Table 3.

**Table 3:** Binary logistic regression analysis for attitude assessment.

					Binary logistic regression			
Variable	N^1^	Negative attitude, *N* = 447	Positive attitude, *N* = 1,818	*p*-value^2^	Coefficient	Odds ratio	95% CI	*p*-value
What is your age?	2,265			<0.001				<0.001
≥ 61		10 (2.2%)	9 (0.5%)		—	—	—	
21–30		292 (65%)	952 (52%)		1.5	4.48	1.73, 11.8	
31–40		46 (10%)	270 (15%)		2	7.19	2.66, 19.7	
41–50		1 (0.2%)	9 (0.5%)		2.4	10.6	1.49, 219	
51–60		98 (22%)	578 (32%)		2.2	9.39	3.48, 25.7	
What is your gender?	2,265			0.047				0.13
Female		276 (62%)	1,213 (67%)		—	—	—	
Male		171 (38%)	605 (33%)		−0.18	0.84	0.66, 1.05	
What is your marital status?	2,265			0.6				0.1
Divorced		9 (2.0%)	25 (1.4%)		—	—	—	
Married		186 (42%)	756 (42%)		0.83	2.29	0.95, 5.10	
Single		252 (56%)	1,037 (57%)		0.94	2.57	1.06, 5.80	
How many members do you have in your family?	2,265			0.012				0.041
≤ 2		35 (7.8%)	80 (4.4%)		—	—	—	
≥ 4		300 (67%)	1,260 (69%)		0.65	1.92	1.16, 3.14	
3		112 (25%)	478 (26%)		0.59	1.81	1.05, 3.08	
What type of institution do you currently work in?	2,265			0.7				0.2
Clinic		22 (4.9%)	106 (5.8%)		—	—	—	
Hospital		307 (69%)	1,250 (69%)		−0.5	0.6	0.34, 1.04	
Private Practice		118 (26%)	462 (25%)		−0.54	0.58	0.31, 1.04	
What is your monthly household income?	2,265			<0.001				<0.001
≤ 50,000		85 (19%)	214 (12%)		—	—	—	
≥ 80,000		37 (8.3%)	331 (18%)		1.2	3.19	2.05, 5.02	
51,000-80,000		51 (11%)	222 (12%)		0.39	1.48	0.97, 2.26	
Prefer Not to disclose		274 (61%)	1,051 (58%)		0.09	1.09	0.80, 1.49	
What is your Employment Status?	2,265			0.007				0.8
Contract		229 (51%)	781 (43%)		—	—	—	
Permanent Employee		195 (44%)	932 (51%)		−0.02	0.98	0.75, 1.27	
Visiting		23 (5.1%)	105 (5.8%)		0.15	1.17	0.70, 2.01	
What is your role in the Healthcare setting?	2,265			>0.9				>0.9
Administration		37 (8.3%)	137 (7.5%)		—	—	—	
Nurse		90 (20%)	376 (21%)		0.16	1.17	0.73, 1.88	
Other		50 (11%)	208 (11%)		0.08	1.08	0.64, 1.81	
Physician		44 (9.8%)	176 (9.7%)		0.09	1.09	0.64, 1.85	
Technician		226 (51%)	921 (51%)		0.04	1.05	0.68, 1.57	
Knowledge	2,265			<0.001				<0.001
knowledgeable		237 (53%)	1,498 (82%)		—	—	—	
Not knowledgeable		210 (47%)	320 (18%)		−1.4	0.24	0.19, 0.30	
Practice	2,265			0.9				>0.9
Good Practice		418 (94%)	1,704 (94%)		—	—	—	
Poor Practice		29 (6.5%)	114 (6.3%)		−0.03	0.97	0.63, 1.55	

1 n (%).

2 Fisher’s exact test; Pearson’s Chi-squared test.

#### 3.4.3 Practice

The practice suggested diverse influences across different factors. Among, age demonstrated no significant influence on practice habits, as indicated by the high odds ratios with indeterminate confidence intervals (CIs), suggesting inconsistent effects across different age groups. For example, participants aged 21–30 and 31–40 showed extremely high odds ratios (ORs of 3,416,725 and 3,601,871 respectively), but these figures likely indicated data anomalies or calculation errors due to small sample sizes in certain age brackets.

Gender seemed to have a negligible effect on hygiene practices, with males and females exhibiting similar probabilities of adhering to good practices (OR = 0.95, 95% CI = 0.65, 1.36, *p*-value = 0.8). Marital status also showed minimal impact, although married participants showed a slightly higher inclination toward better practices (OR = 2.29, 95% CI = 0.95, 5.10, *p*-value = 0.071). Family size and the type of institution worked in did not significantly affect hand hygiene practices, with similar adherence rates across groups with different family sizes and those working in clinics, hospitals, or private practices. Household income showed some variation, with those earning between 51,000–80,000 showing a moderate increase in good practice adherence (OR = 2.17, 95% CI = 1.09, 4.52, *p*-value = 0.2), as shown in Table 4.

**Table 4:** Binary logistic regression analysis for practice assessment.

					Binary logistic regression			
Variable	N^1^	Good practice, *N* = 2,122	Poor practice, *N* = 143	*p*-value^2^	Coefficient	Odds ratio	95% CI	*p*-value
What is your age?	2,265			>0.9				0.3
≥ 61		19 (0.9%)	0 (0%)		—	—	—	
21–30		1,166 (55%)	78 (55%)		0.57	1.78	0.68, 2.93	
31–40		294 (14%)	22 (15%)		0.36	2.57	1.09, 4.52	
41–50		10 (0.5%)	0 (0%)		0.91	1.72	0.91, 3.11	
51–60		633 (30%)	43 (30%)					
What is your gender?	2,265			0.7				0.8
Female		1,393 (66%)	96 (67%)		—	—	—	
Male		729 (34%)	47 (33%)		−0.05	0.95	0.65, 1.36	
What is your marital status?	2,265			0.3				0.071
Divorced		34 (1.6%)	0 (0%)		—	—	—	
Married		877 (41%)	65 (45%)		15	2.29	0.95, 5.10	
Single		1,211 (57%)	78 (55%)		15	2.57	1.06, 5.80	
How many members do you have in your family?	2,265			>0.9				0.6
≤ 2		108 (5.1%)	7 (4.9%)		—	—	—	
≥ 4		1,460 (69%)	100 (70%)		0.11	1.11	0.50, 2.85	
3		554 (26%)	36 (25%)		−0.13	0.88	0.37, 2.36	
What type of institution do you currently work in?	2,265			0.3				0.2
Clinic		120 (5.7%)	8 (5.6%)		—	—	—	
Hospital		1,466 (69%)	91 (64%)		−0.17	0.84	0.40, 2.04	
Private Practice		536 (25%)	44 (31%)		0.18	1.2	0.54, 2.99	
What is your monthly household income?	2,265			0.2				0.2
≤ 50,000		286 (13%)	13 (9.1%)		—	—	—	
≥ 80,000		347 (16%)	21 (15%)		0.32	1.38	0.68, 2.93	
51,000-80,000		249 (12%)	24 (17%)		0.78	2.17	1.09, 4.52	
Prefer Not to disclose		1,240 (58%)	85 (59%)		0.48	1.62	0.91, 3.11	
What is your Employment Status?	2,265			0.2				0.2
Contract		954 (45%)	56 (39%)		—	—	—	
Permanent Employee		1,052 (50%)	75 (52%)		0.25	1.28	0.85, 1.94	
Visiting		116 (5.5%)	12 (8.4%)		0.62	1.86	0.90, 3.60	
What is your role in the healthcare setting?	2,265			0.9				0.7
Administration		166 (7.8%)	8 (5.6%)		—	—	—	
Nurse		438 (21%)	28 (20%)		0.14	1.15	0.52, 2.81	
Other		240 (11%)	18 (13%)		0.36	1.43	0.62, 3.61	
Physician		205 (9.7%)	15 (10%)		0.46	1.58	0.66, 4.03	
Technician		1,073 (51%)	74 (52%)		0.36	1.43	0.71, 3.28	
Knowledge	2,265			0.4				0.3
Knowledgeable		1,630 (77%)	105 (73%)		—	—	—	
Not knowledgeable		492 (23%)	38 (27%)		0.24	1.27	0.84, 1.90	
Attitude	2,265			0.9				0.9
Negative Attitude		418 (20%)	29 (20%)		—	—	—	
Positive Attitude		1,704 (80%)	114 (80%)		−0.03	0.97	0.63, 1.54	

1 n (%).

2 Fisher’s exact test; Pearson’s Chi-squared test.

## 4 Discussion

The responses from 2,265 healthcare workers provided critical insights into their hand hygiene practices, attitudes, and knowledge, along with detailed demographic information. The cohort was predominantly younger, with 55% aged between 21 and 30 years and 30% aged between 51 and 60 years, representing a diverse age range. A significant majority of participants were female (66%) and single (57%), with 69% coming from family units of four or more. Most were employed in hospitals (69%), while 58% preferred not to disclose their income. The study revealed that 77% of participants demonstrated comprehensive hand hygiene knowledge, 80% held positive attitudes toward hand hygiene, and 94% adhered to good practices. These findings highlight the strong foundation of hand hygiene awareness and compliance among healthcare workers, which is crucial for effective infection control and informs the development of targeted interventions to further enhance these practices (28, 29). The need for ongoing education is crucial, as knowledge directly impacts adherence to hand hygiene protocols, which can decrease HAIs by 30 to 50%. In contrast, a study in Lahore revealed lower knowledge and practice scores among healthcare personnel, particularly nurses, compared to the high compliance observed in the current study (30). This discrepancy might be attributed to demographic differences and variations in institutional training programs. The higher compliance in the current study May be due to the structured educational efforts and monitoring, which were less evident in the Lahore study. Moreover, a qualitative assessment in Guinea emphasized the impact of the Ebola outbreak on improving hand hygiene practices temporarily, suggesting that sustained improvements require long-term commitment and regular training (31). This study reinforces the notion that fear-driven compliance is temporary, underscoring the need for continuous education and motivation beyond immediate health crises.

The current study reveals mixed responses regarding attitudes toward hand hygiene, with a significant proportion of healthcare workers unsure about proper hand hygiene techniques despite understanding their importance for safety. This reflects findings in a study conducted at a Vietnamese university, where despite high awareness, actual compliance with hand hygiene protocols was inconsistent, stressing the need for reinforced training and clearer guidelines (32, 33). The uncertainty expressed by participants in the current study underscores a similar gap in confidence that could be bridged through enhanced educational outreach.

Regarding practices, while a strong commitment to promoting hand hygiene was noted, inconsistencies in glove use and hand washing between patient contacts indicate room for improvement. This aligns with the situation described at Faranah Regional Hospital, Guinea, where despite previous improvements during the Ebola crisis, a decline in strict adherence to hand hygiene practices was observed post-crisis (31). This suggests that ongoing motivation and regular audits are crucial to maintaining high standards of hygiene practices. Moreover, the strong support for regular hand hygiene reminders and the acknowledged need for enhanced managerial support and resources in the current study reflect sentiments from research in Lahore, which indicated that better resource allocation and supportive management could foster improved hygiene practices among healthcare workers (30). The desire for more training sessions and clearer guidelines mirrors the global need for continuous improvement in hand hygiene education to effectively combat HAIs (34).

The current study indicated that 62% of healthcare workers recognize alcohol-based hand sanitizers as a viable alternative when soap and water are not available. This aligns with the CDC’s recommendation that hand sanitizers with at least 60% alcohol can be effective in reducing microbes on hands in certain situations (35, 36). However, it is also noted that sanitizers do not eliminate all types of germs, which May explain why 39% were unsure about the link between improper hygiene and HAIs (37). The uncertainty among 36% of participants about hand hygiene’s role in curbing non-influenza respiratory illnesses contrasts with WHO guidelines, which emphasize hand hygiene as a crucial measure to prevent the spread of infections (38). The 58% acknowledgment rate among healthcare workers could be improved through targeted education, reinforcing the importance of hand hygiene as supported by global health authorities (9, 39, 43).

Interestingly, the practice of washing hands for a minimum of 20 s is strongly supported by 84% of participants, which is in line with the CDC’s handwashing guidelines (40).

In our study, healthcare workers exhibited a robust knowledge base and positive attitudes toward hand hygiene, with 77% demonstrating strong knowledge and 80% showing positive attitudes. These results can be contrasted with findings where healthcare workers who participated in formal training sessions at their facilities reported higher knowledge and attitude scores toward influenza vaccination. This correlation suggests that structured educational programs are effective in enhancing understanding and shaping positive health behaviors (14, 44). In our study, despite a high level of knowledge across the board, there was no direct correlation between knowledge levels and the actual practice of hand hygiene, which is akin to the observed trend where higher knowledge about influenza did not necessarily predict vaccination rates.

Healthcare workers’ engagement in hand hygiene practices demonstrates significant adherence, with variances across specific behaviors and settings. A notable 93% of healthcare workers advocate for hand hygiene among colleagues and patients, emphasizing a strong commitment to these practices (45). However, about 32% of workers still engage in potentially contaminating behaviors like touching their face or personal items during shifts (41). Regarding procedural hygiene, 63% consistently change gloves and wash hands between different patient contacts, but 28% do not follow this protocol, suggesting a need for stricter adherence and regular audits, as only 42% participate in regular hygiene audits (42). The high participation rate in annual influenza vaccination at 82% indicates a proactive approach toward infection prevention. However, the regular use of hand sanitizer, endorsed by 79% of the workers, highlights its acceptance as an essential hygiene tool when soap and water are not available (31). This data underscores the importance of consistent training, availability of hygiene materials, and mandatory hygiene audits to improve overall hand hygiene compliance within healthcare settings.

The logistic regression analysis provides valuable insights into the factors influencing hand hygiene knowledge, attitudes, and practices among healthcare workers. For example, the OR of 4.48 for healthcare workers aged 21–30 years indicates that younger professionals are significantly more likely to hold positive attitudes toward hand hygiene compared to their older counterparts. This suggests that targeted educational interventions could be particularly effective if focused on sustaining and further enhancing these positive attitudes in younger workers. Additionally, the OR of 3.19 for participants with higher income levels (≥80,000) suggests a strong association between socioeconomic status and positive attitudes toward hand hygiene. This finding underscores the need for tailored strategies that consider the economic context of healthcare workers, potentially addressing disparities in resource availability and access to training.

Moreover, the OR of 1.24 for good practices associated with higher knowledge levels reinforces the critical role of continuous education in promoting adherence to hand hygiene protocols. These practical implications highlight the importance of not only maintaining but also intensifying training and support mechanisms across various demographic groups to ensure consistent and effective hand hygiene practices. The results provide actionable insights for policymakers and healthcare administrators to design and implement interventions that are both demographically sensitive and contextually appropriate, ultimately aiming to reduce healthcare-associated infections and improve patient outcomes (46).

While our study shows strong hand hygiene practices among healthcare workers, several barriers May hinder consistent adherence. Key challenges include limited access to resources like hand sanitizers, especially in lower-income settings, and the perception of hand hygiene as a lower priority amid heavy workloads. Additionally, gaps in confidence regarding proper hand hygiene techniques can lead to inconsistent application. To overcome these barriers, it is essential to ensure consistent access to hygiene supplies and to integrate hand hygiene education into ongoing training programs. Emphasizing the importance of hand hygiene through regular reminders and audits can also help embed these practices into daily routines, making them an integral part of patient care. Addressing these barriers is critical for reducing healthcare-associated infections and improving overall patient outcomes.

In addition, this study has a few limitations, the study’s cross-sectional design limits causal inference, and reliance on self-reported data May introduce response bias, potentially skewing results. The dissemination method via email and social media May have excluded some potential participants with limited digital access, affecting sample representativeness. Broad inclusion criteria and linguistic limitations could also introduce selection biases, affecting the diversity and accuracy of the data. Additionally, the use of stratified random sampling does not ensure a sample perfectly reflective of the broader healthcare worker population in Hubei, China, and data integrity issues could impact the reliability of the findings. Notwithstanding, to our knowledge, this is the first KAP study to assess post-pandemic influenza control among healthcare workers in Hubei, China.

## 5 Conclusion

The current study highlights significant insights into the hand hygiene behaviors of healthcare workers, showing a strong foundation in KAP essential for infection control. While the majority of respondents demonstrated good knowledge and compliance with hand hygiene protocols, gaps in confidence and perceptions indicate a need for enhanced educational programs. The results suggest that reinforcing the understanding of hand hygiene’s impact on preventing respiratory illnesses and HAIs could further strengthen practices. Additionally, the data reveals a disparity in the support from management to adhere to hand hygiene standards, highlighting an area for institutional improvement. Regular reminders and comprehensive training sessions could serve to boost both knowledge and the proper implementation of hygiene practices. Addressing these gaps through targeted interventions could significantly enhance the effectiveness of hand hygiene measures, ensuring a safer healthcare environment and reducing the risk of infection transmission.

## Data Availability

The raw data supporting the conclusions of this article will be made available by the authors, without undue reservation.
